# Deep Learning Indoor Positioning for Connected Aircraft Cabins: A ResNet Approach with Real-World Validation

**DOI:** 10.3390/s26051569

**Published:** 2026-03-02

**Authors:** Paul Schwarzbach, Muhammad Ammad, Michael Schultz, Oliver Michler

**Affiliations:** 1 Transport Systems Information Technology, TUD Dresden University of Technology, 01062 Dresden, Germany; muhammad.ammad@tu-dresden.de (M.A.); oliver.michler@tu-dresden.de (O.M.); 2 Air Traffic Concepts, University of the Bundeswehr Munich, 85577 Neubiberg, Germany; michael.schultz@unibw.de

**Keywords:** ResNet architecture, likelihood grid maps, Bluetooth Low Energy (BLE), Ultra-Wide Band (UWB), sensor fusion, multilateration

## Abstract

Indoor positioning in aircraft cabins presents fundamental challenges arising from severe multipath propagation, non-line-of-sight conditions, and metallic fuselage geometry that degrade radio-based positioning methods. This study validates a residual neural network (ResNet) based deep learning approach for aircraft cabin localization through real-world measurements in an A320 cabin mockup. The methodology employs dual-technology ranging measurements from Ultra-Wideband and Bluetooth Low Energy, transforming range observations into spatial likelihood representations processed by a ResNet. Experimental validation encompasses 19 distributed measurement positions, evaluated against three baseline methods: iterative least squares, robust least squares with Huber loss, and Bayesian grid filtering. ResNet achieved an overall median positioning error of 0.177 m, achieving lower positioning errors than all three baseline methods. Results confirm that likelihood-based neural network positioning is viable for operational aircraft cabin deployment while identifying performance dependencies on anchor visibility, measurement height, and propagation conditions. The original data is openly available.

## 1. Introduction

Indoor positioning systems serve critical functions across application domains, including navigation, asset tracking, and location-based services in complex environments such as shopping malls, airports, hospitals, and industrial settings [1,2]. Radio-based indoor positioning systems use wireless communication technologies such as Ultra-Wideband (UWB), WiFi, Bluetooth Low Energy (BLE), and emerging cellular systems to derive position estimates from signal measurements [3,4]. These solutions face fundamental challenges arising from signal interference, multipath propagation, and environmental complexities that severely degrade positioning accuracy [5], as conventional positioning methods exhibit high sensitivity to measurement errors introduced by the propagation environment [2]. Machine learning (ML) improves indoor positioning performance beyond traditional approaches by modeling complex patterns [6,7]. Data-driven methods can be categorized into direct ML positioning, which estimates locations from raw sensor data through trained models, and ML-assisted positioning, which integrates learning components for tasks such as non-line-of-sight (NLOS) identification, ranging error correction, and measurement filtering [8]. Deep neural networks, including convolutional neural networks (CNNs) and Long Short-Term Memory (LSTM) architectures, extract discriminative features from radio measurements without requiring explicit channel models [9,10,11,12]. These learning-based approaches can implicitly capture complex mappings between radio signal characteristics and spatial locations, making them well-suited to challenging propagation environments.

The aircraft cabin represents a particularly demanding test case for indoor positioning technologies. The elongated fuselage geometry, densely packed seating, metallic structural elements, and passenger presence create severe multipath propagation with frequent NLOS conditions [13,14]. These phenomena introduce substantial ranging errors, particularly positive biases from reflected and diffracted signal paths, degrading the geometric dilution of precision upon which conventional positioning algorithms depend.

Deep learning approaches address NLOS conditions through learned representations of channel characteristics. For UWB-based systems, CNNs and hybrid architectures have achieved NLOS classification accuracies of up to 97% and ranging error reductions of 30–40% [15,16,17]. ResNet architectures train very deep networks through skip connections that mitigate gradient degradation [18]. Prior work developed ResNet-based positioning using simulation-generated training data, where deterministic ray-tracing combined with statistical error models provided large-scale datasets capturing both geometric propagation effects and stochastic measurement uncertainties [19,20,21,22]. However, real-world validation is needed to confirm that learned representations generalize to operational conditions.

Beyond technical challenges, accurate cabin localization addresses pressing operational needs. Aircraft turnaround delays cost airlines an estimated $30 per ground minute [23], with boarding representing the least predictable turnaround phase due to its dependence on individual passenger behavior [24]. Simulation studies have shown that optimized boarding sequences can reduce boarding times by up to 50% compared to random boarding [25], motivating research into real-time cabin monitoring for adaptive boarding control [24]. The COVID-19 pandemic further highlighted this need, as physical distancing requirements nearly doubled boarding durations [26,27], demonstrating the operational value of spatial awareness for cabin management. Cabin localization extends beyond verifying whether passengers have boarded. Boarding pass scanning at the aircraft door provides binary presence confirmation but yields no spatial information once a passenger enters the cabin. During the boarding phase, which typically lasts 15 to 45 min and covers the primary time window of the proposed localization approach, passengers move through the aisle, stow luggage, and transition between rows, making assigned seat positions an unreliable proxy for actual location. The connected cabin concept requires location awareness for multiple object categories [13]: safety equipment such as life vests and emergency supplies, whose presence must be manually verified by cabin crew during pre-flight checks due to documented removal by passengers; service equipment, including catering trolleys and cleaning carts during turnaround operations; and crew positioning during boarding and emergency scenarios. While boarding status (on or off the aircraft) can be verified through the passenger manifest, position within the cabin is what enables process optimization: real-time boarding progress prediction for target off-block time estimation [24], detection of aisle congestion, and quantification of boarding completion percentage for ground handling coordination. Complementary to tag-based localization, passive radio sensing can detect seat occupancy through analysis of multipath propagation changes without requiring passengers to carry dedicated devices [28].

**Figure 1:**
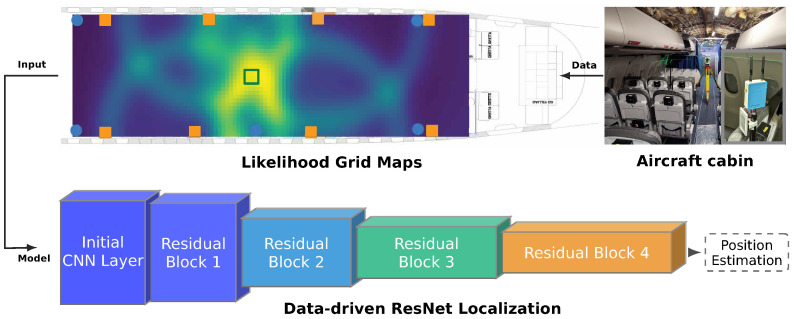
System overview of the ResNet-based position estimation using likelihood grid maps derived from BLE and UWB measurements in an aircraft cabin.

This paper validates ResNet-based positioning through real-world measurements in a full-scale A320 cabin mockup (cf. Figure 1). We build upon prior simulation-based development [22] and evaluate likelihood-based neural network localization using dual-technology ranging measurements from UWB Time-of-Arrival and BLE-compliant phase-based systems. Range observations are transformed into spatial likelihood representations serving as ResNet inputs. Performance evaluation encompasses 19 systematically distributed measurement positions compared against three baseline methods: iterative least squares, robust least squares with Huber loss, and probabilistic grid filtering. This comparative analysis quantifies positioning accuracy under severe multipath and NLOS conditions characteristic of aircraft cabin environments. Regarding hardware feasibility, the methodology is independent of tag form factor. Modern smartphones increasingly integrate both UWB and BLE: since 2019, flagship devices from major manufacturers include UWB chips alongside near-ubiquitous BLE connectivity [29]. Digital boarding passes on smartphones could integrate positioning via UWB and BLE APIs now available on Android and iOS, and commercially available card-form-factor UWB tags offer an alternative for passengers without compatible devices. The primary operational scenarios, boarding and turnaround, occur on the ground before airplane mode restrictions apply, when passenger devices are fully active. Beyond passenger-carried devices, compact tags can be attached to safety equipment, service trolleys, or other cabin assets to enable inventory tracking and operational awareness. The anchors, e.g., UWB and BLE transceivers integrated into the cabin wall panelling or embedded in existing cabin electronics such as in-flight entertainment displays, represent fixed infrastructure. The positioning algorithms process range measurements identically regardless of whether they originate from smartphones, card tags, asset-mounted devices, or dedicated hardware, as used in this study.

### 1.1. Related Work

The scope of this work spans multiple domains, including operational applications requiring location awareness, radio-based indoor positioning, and machine learning.

#### 1.1.1. Indoor Positioning for Operational Applications

Aircraft turnaround optimization has been the subject of extensive research, with particular focus on passenger boarding procedures. Studies have investigated optimal boarding strategies considering operational constraints such as the seat load factor, group composition, and baggage volume [23,24,25,30]. A fundamental limitation of current approaches is the absence of real-time cabin state information. Aircraft cabins typically lack sensor infrastructure, preventing optimization strategies from adapting to actual conditions and limiting the effectiveness of boarding sequence planning [31]. The COVID-19 pandemic has further emphasized this limitation, as requirements for physical distancing during boarding significantly extended turnaround times, with boarding durations nearly doubling when distance rules are strictly applied [26,27].

Indoor positioning systems (IPS) represent a technological solution to address these operational challenges through real-time location awareness. IPS have been studied extensively, driven by the proliferation of location-based services and the limitations of satellite navigation in indoor environments [1,5].

Surveys of IPS technologies address trade-offs between accuracy, coverage, infrastructure requirements, and cost [3,4], categorizing positioning methods into geometric approaches such as multilateration and fingerprinting techniques that match signal characteristics against location-tagged databases [1,2,32]. UWB is well suited for high-precision positioning: its large bandwidth provides fine temporal resolution for timing-based measurements and inherent resistance to interference, enabling centimeter-level ranging under favorable LOS conditions [1]. Accuracy degrades in NLOS scenarios, where obstructions introduce positive range biases through diffraction, reflection, and scattering [20].

The aircraft cabin presents distinct challenges for radio-based positioning. The metallic fuselage creates a highly reflective environment with strong multipath propagation, while the elongated geometry and densely arranged seats produce complex visibility conditions between anchors and mobile devices [13]. Empirical channel measurements have characterized cabin-specific propagation phenomena, including the effect of human presence on UWB propagation [14,33], and Cogalan et al. [34] developed measurement-based channel models providing parameters for stochastic simulation of realistic cabin conditions.

For localization, Neuhold et al. [35] demonstrated the feasibility of UWB ranging in aircraft while highlighting challenges posed by cabin geometry. Karadeniz et al. [36] achieved submeter UWB positioning accuracy, and Geyer and Schupke [37] combined UWB measurements with ML for onboard localization, showing improved accuracy through learned error compensation. The integration of deterministic ray-tracing simulation with stochastic error modeling has been proposed to enable systematic evaluation of positioning algorithms under realistic cabin conditions [19,38].

#### 1.1.2. Data-Driven Localization and Deep Learning Approaches

ML-based indoor positioning spans traditional algorithms and deep learning architectures that learn feature representations directly from sensor data [6,7]. These methods fall into direct ML positioning, which estimates locations from raw data using trained models, and ML-assisted positioning, which integrates learning into conventional frameworks for NLOS identification, ranging error correction, and measurement filtering [8,39,40]. Both approaches have demonstrated lower positioning errors than model-based methods, particularly in challenging propagation environments. CNNs extract spatial features effectively from structured inputs, suiting them for positioning tasks. Kim et al. [9] presented a scalable deep neural network for multi-building and multi-floor WiFi fingerprinting with substantial error reductions. Transforming channel state information into image-like representations enables CNN-based indoor localization, where networks learn location-discriminative patterns in frequency and spatial domains [11,12]. For UWB systems, deep learning has been applied to NLOS identification, and ranging error mitigation. Yang et al. [15] demonstrated reliable UWB localization in NLOS scenes through spatial–temporal feature learning from channel impulse responses. CNN, recurrent, and hybrid architectures have achieved classification accuracies up to 97% and ranging error reductions of 30–40% in NLOS scenarios [16,17,41]. ResNet architectures suit positioning tasks because skip connections enable training of deep networks while mitigating gradient degradation [18].

### 1.2. Scope and Contribution

This work bridges simulation-based development and operational deployment of deep learning positioning systems through systematic real-world validation in aircraft cabin environments. We provide an empirical validation of deep learning-based positioning in a real aircraft cabin environment, establishing performance benchmarks and identifying the propagation phenomena that govern accuracy in this demanding setting. While prior research has demonstrated ResNet-based localization using synthetic data [22], the generalization of learned representations to actual radio propagation conditions with hardware-induced errors, environmental variability, and measurement outliers remains unestablished. The contributions of this study are threefold.

First, we validate likelihood-based ResNet positioning through a systematic measurement campaign in a full-scale A320 cabin mockup, providing empirical performance characterization across 19 reference positions, with ground truth established via geodetic surveying. Second, we conduct comparative evaluation against baseline methods (iterative least squares, robust least squares, Bayesian grid filtering) using dual-technology ranging measurements from UWB and BLE-compliant systems, enabling assessment of data-driven approaches relative to conventional parametric and non-parametric methods. Third, we characterize spatial performance heterogeneity and ranging error mechanisms to identify the specific propagation phenomena and geometric factors that influence positioning accuracy in confined metallic environments with severe multipath propagation.

The rest of the paper is structured as follows. After the introduction in Section 1, Section 2 details the experimental methodology, including radio technologies, measurement infrastructure, baseline positioning algorithms, and ResNet architecture. Section 3 presents the ranging and positioning results. Section 4 concludes the paper by interpreting the results through an analysis of error mechanisms, spatial patterns, and methodological tradeoffs; summarizing the main findings; and identifying directions for future research.

## 2. Materials and Methods

### 2.1. Radio Technologies for Indoor Positioning

The dataset employed in this study comprises ranging measurements from two radio technologies: UWB and BLE. The dual-technology approach enables systematic evaluation of data-driven localization across different measurement characteristics and cost–deployment trade-offs. The UWB system operates in accordance with IEEE 802.15.4a specifications, employing Two-Way Ranging (TWR) to eliminate clock synchronization requirements between devices [42,43,44]. The selection of UWB for this study is motivated by its demonstrated suitability for high-precision positioning in challenging environments and its widespread adoption in aircraft cabin localization research [35,36,37].

BLE provides a complementary technology with distinct advantages in widespread device availability and infrastructure cost, as it has achieved near-ubiquitous deployment in consumer electronics (smartphones, tablets, and wearable devices) [3]. This market penetration enables deployment scenarios where passengers’ personal devices function as mobile nodes without requiring specialized hardware, potentially reducing both infrastructure investment and operational complexity. For ranging, BLE uses phase-based ranging, which exploits the relationship between the signal phase and propagation distance through multi-carrier phase differencing. The approach measures phase differences across multiple frequency carriers in the 2.4 GHz ISM band, with distance estimates derived through inverse Fourier transform processing that converts phase differences to time delays [45,46,47]. Recent standardization through BLE Channel Sounding has formalized these capabilities, with commercial implementations demonstrating sub-meter ranging accuracy in indoor settings [48]. The inclusion of BLE ranging addresses the practical deployment consideration that specialized UWB infrastructure may be impractical or cost-prohibitive in retrofit scenarios, while BLE uses existing device ecosystems. However, the narrower bandwidth of BLE compared to UWB provides a reduced temporal resolution for multipath discrimination [46].

### 2.2. Data Acquisition

**Figure 2:**
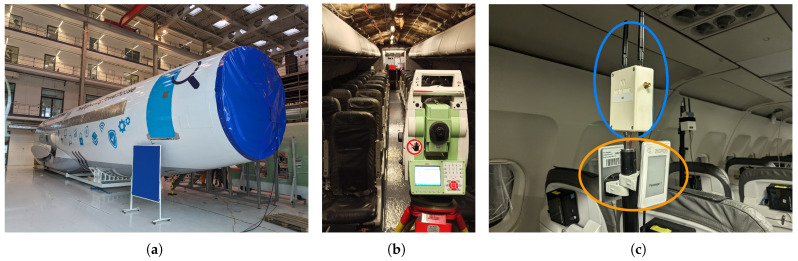
Experimental environment and used hardware: (**a**) connected cabin mockup, (**b**) total station and (**c**) BLE PBR-compliant tags (blue) and UWB tags (orange).

To validate the proposed localization framework with real-world measurements, we conducted an experimental campaign in a realistic aircraft cabin environment. The data acquisition was performed in an aircraft cabin mockup at the Center of Applied Aeronautical Research in Hamburg, Germany. The mockup represents a single-aisle commercial aircraft cabin configuration with 10 seat rows accommodating 60 passenger seats in total, representing a partial A320 cabin (cf. Figure 2).

#### 2.2.1. Deployed Positioning Systems and Reference System

Two distinct positioning systems were deployed in the cabin mockup. The BLE system employs a proprietary phase-based ranging approach developed by Metirionic GmbH (Dresden, Germany) [49]. This system, while BLE-compatible, uses a custom ranging implementation rather than the standardized BLE Channel Sounding [48]. At the time of data acquisition (July 2023), the Channel Sounding specification had only recently been released, and commercial hardware implementations were not yet widely available. The Metirionic system therefore represents an early implementation of BLE phase-based ranging concepts that were subsequently formalized in the official specification. The UWB system, provided by ZigPos GmbH (Dresden, Germany), operates in UWB Channel 2, conforming to IEEE 802.15.4a specifications [42] and implements TWR [50].

To establish high-precision reference coordinates for anchor and tag positions, we employed an optical Leica total station [51]. The total station enables millimeter-level accuracy in three-dimensional coordinate determination through combined angular and distance measurements. Prior to data collection, all anchor node positions were surveyed and recorded in a common coordinate system. For each tag measurement position, the total station was used to survey the ground-truth position against which positioning system estimates could be evaluated. The reference measurement accuracy far exceeds the anticipated performance of all evaluated positioning systems, so observed errors reflect true system performance.

#### 2.2.2. Anchor Deployment and Measurement Positions

**Figure 3:**
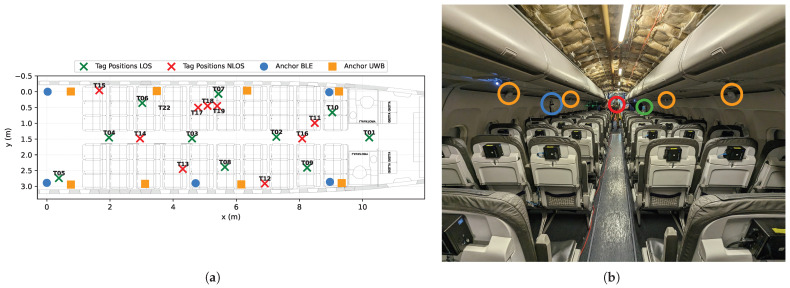
Measurement infrastructure: (**a**) overview map of device positions within the cabin mockup, showing anchor nodes (orange triangles: UWB; blue triangles: BLE) and tag positions (green circles: LOS to majority of anchors; red circles: NLOS-dominated). (**b**) Installed infrastructure color-coded by system (green: mobile tags; orange: UWB anchors; blue: BLE anchors; red: total station). The deployment spans 10 seat rows in a single-aisle configuration.

The positioning infrastructure consisted of stationary anchor nodes mounted at fixed locations throughout the cabin mockup. Anchor nodes were positioned along the transition between the cabin sidewall and the overhead luggage compartments, distributed along both sides of the cabin over the available length (cf. Figure 3). For the BLE system, five anchor nodes were deployed with approximately equal spacing along the cabin length. The UWB systems used eight anchor nodes.

Mobile tag devices were positioned within the cabin environment to systematically evaluate positioning performance under diverse geometric and propagation conditions. Tag positions were distributed across multiple seat rows, encompassing locations along the aisle, within seat positions (window, middle, aisle seats), and at varying distances from anchor nodes. This constellation was designed to capture the full range of positioning scenarios likely to occur during operational passenger tracking, including both favorable (near-aisle, strong LOS) and challenging (window seats, obstructed propagation) conditions. To investigate the impact of visibility on accuracy, measurements were conducted with tags placed at different heights:

- Seat surface (approximately 0.45 m above floor);
- Beneath the seat (approximately 0.25 m above floor, representing potential applications for baggage tracking or under-seat wearable devices);
- Above the seat (approximately 1.1 m above floor, representing passenger-worn devices such as smartphones or wearable sensors).

Tags were mounted on adjustable tripods to enable precise vertical positioning and stable measurements at each location (Figure 3a). The tripod mounting ensured consistent tag orientation and eliminated motion-induced measurement variations during data collection.

### 2.3. Baseline Positioning Methods

To evaluate the performance of the proposed ResNet approach, three baseline positioning methods are compared: iterative least squares (ILS) using the Gauss–Newton method, robust least squares (RLS) employing the Huber loss function, and probabilistic Bayesian grid filtering (BGF). These state-of-practice methods provide benchmarks for assessing data-driven localization. The progression from parametric to robust parametric to non-parametric methods enables systematic evaluation of how different uncertainty representations handle the complex error characteristics observed in aircraft cabin environments.

#### 2.3.1. Iterative Least Squares (ILS)

ILS solves the nonlinear multilateration problem by minimizing the sum of squared residuals. Given *N* anchor positions $a_{i}={\left(x_{i},y_{i}\right)}^{⊤}$ and corresponding range measurements ${\tilde{r}}_{i}$ (i=1,…,N), the objective is to estimate the tag position $p={\left(x,y\right)}^{⊤}$ with:

(1) $$p^{*}=\arg\min_{p}\sum_{i=1}^{N}{\left({\tilde{r}}_{i}-{∥p-a_{i}∥}_{2}\right)}^{2}$$

ILS iteratively refines the estimate, with the update at iteration *k* computed as

(2) $$p^{\left(k+1\right)}=p^{\left(k\right)}-{\left(J^{⊤}J\right)}^{-1}J^{⊤}r^{\left(k\right)}$$

where $r^{\left(k\right)}$ is the residual vector with elements $r_{i}^{\left(k\right)}={\tilde{r}}_{i}-{∥p^{\left(k\right)}-a_{i}∥}_{2}$, and J is the Jacobian matrix, with elements

(3) $${\left[J\right]}_{ij}=\frac{{\left[p^{\left(k\right)}\right]}_{j}-{\left[a_{i}\right]}_{j}}{∥p^{\left(k\right)}-a_{i}{∥}_{2}}$$

where ${\left[\cdot \right]}_{j}$ denotes the *j*-th component of the vector. The iterative process continues until convergence is achieved ($∥p^{\left(k+1\right)}-p^{\left(k\right)}∥<\epsilon$) or the maximum number of iterations is reached. This method has been extensively applied in positioning systems [52,53] and serves as a fundamental baseline due to its computational efficiency and well-understood convergence properties.

#### 2.3.2. Robust Least Squares (RLS)

RLS addresses the sensitivity of standard least squares to outlier measurements by employing the Huber loss function, which reduces the influence of measurements exhibiting large residuals [54,55]. The Huber loss function $\rho_{H}\left(\cdot \right)$ is defined as

(4) $$\rho_{H}\left(e\right)=\left\{\begin{array}{cc} \frac{1}{2}e^{2} & \mathrm{if}\;|e|\leq \delta \\ \delta |e|-\frac{1}{2}\delta^{2} & \mathrm{if}\;|e|>\delta \end{array}\right.$$

where *e* denotes the residual, and δ is the threshold parameter that determines the transition between quadratic and linear penalty. The estimate is obtained by minimizing

(5) $$p^{*}=\arg\min_{p}\sum_{i=1}^{N}\rho_{H}\left({\tilde{r}}_{i}-{∥p-a_{i}∥}_{2}\right)$$

This loss function exhibits quadratic behavior for residuals below δ but transitions to linear growth for larger residuals, thereby down-weighting outliers. This approach has shown improved performance in the presence of contaminated measurements [54,56].

#### 2.3.3. Bayesian Grid Filter (BGF)

BGF employs non-parametric state estimation to explicitly address non-Gaussian error distributions and multimodal likelihoods that frequently arise in indoor environments [57,58]. The continuous state space is discretized into a finite set of grid cells $G=\{g_{\left(1,1\right)},g_{\left(1,2\right)},\ldots ,g_{\left(M_{x},M_{y}\right)}\}$, where each grid cell $g_{\left(i,j\right)}$ represents a position hypothesis with associated probability $p_{t}\left(g_{\left(i,j\right)}\right)$ at time step *t*. This representation enables the filter to maintain arbitrary probability distributions without imposing parametric constraints [57]. The estimation process follows a recursive Bayesian structure. The prediction step incorporates system dynamics through transition probabilities:

(6) $${\bar{p}}_{t}\left(g_{\left(i,j\right)}\right)=\sum_{m=1}^{M_{x}}\sum_{n=1}^{M_{y}}p_{t-1}\left(g_{\left(m,n\right)}\right)\cdot p\left(g_{\left(i,j\right)}|g_{\left(m,n\right)}\right)$$

where $p(g_{\left(i,j\right)}|g_{\left(m,n\right)})$ models the transition probability between grid cells. The measurement update integrates range observations through Bayes’ rule:

(7) $$p_{t}\left(g_{\left(i,j\right)}\right)=\eta \cdot {\bar{p}}_{t}\left(g_{\left(i,j\right)}\right)\cdot L\left(g_{\left(i,j\right)}\right)$$

where η is the normalization constant and $L(g_{\left(i,j\right)})$ represents the measurement likelihood. For range-based positioning, the likelihood is computed by evaluating residuals between measured ranges ${\tilde{r}}_{i}$ and expected ranges $d_{(i,j),i}={∥g_{\left(i,j\right)}-a_{i}∥}_{2}$ for each anchor:

(8) $$L\left(g_{\left(i,j\right)}\right)=\prod_{k=1}^{N}\exp\left(-\frac{{\left({\tilde{r}}_{k}-d_{(i,j),k}\right)}^{2}}{2\sigma_{k}^{2}}\right)$$

where $\sigma_{k}^{2}$ represents the measurement variance for anchor *k*. The position estimate is extracted as the probability-weighted mean of grid cells within radius ρ around the maximum likelihood estimate:

(9) $$p^{*}=\frac{\sum_{\left(i,j\right)\in B_{\rho }}p_{t}\left(g_{\left(i,j\right)}\right)\cdot g_{\left(i,j\right)}}{\sum_{\left(i,j\right)\in B_{\rho }}p_{t}\left(g_{\left(i,j\right)}\right)}$$

where $B_{\rho }$ defines the set of grid cells within localization radius ρ of the maximum likelihood cell. Implementation details are provided in Schwarzbach et al. [19,59].

### 2.4. Deep-Learning ResNet Localization

The data-driven method in this work uses a ResNet [18] to estimate tag positions from spatial likelihood representations derived from range measurements. Unlike the parametric and robust parametric baselines (Section 2.3), the network directly infers coordinates from measurement-derived spatial features through supervised training. The architecture builds upon prior development [22], here trained and validated exclusively with experimental data from the aircraft cabin mockup.

#### 2.4.1. Likelihood Grid Map Construction

Following the BGF framework (Section 2.3.3), the spatial domain is discretized into a uniform grid G with dimensions $M_{x}\times M_{y}$. For each grid cell $g_{\left(i,j\right)}$ and anchor $a_{k}$, the Gaussian observation model evaluates the compatibility between measured range ${\tilde{r}}_{k}$ and expected range $d_{(i,j),k}={∥g_{\left(i,j\right)}-a_{k}∥}_{2}$, constructing per-anchor likelihood maps $L_{k}^{\left(r\right)}\left(g_{\left(i,j\right)}\right)$. These likelihood maps provide spatially resolved representations of measurement compatibility, with higher values indicating greater likelihoods that the tag occupies the corresponding grid cell. Measurements from *N* anchors yield *N* individual likelihood maps, providing *N* input channels for the neural network.

**Figure 4:**
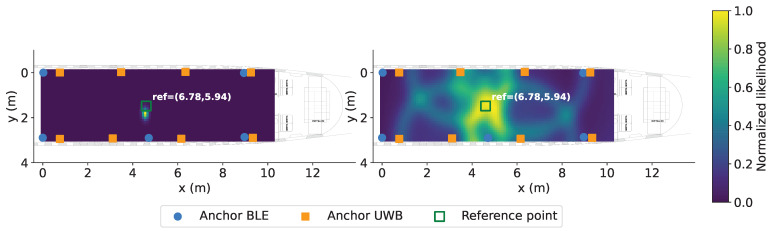
Residual maps of BGF integrated likelihood as product of observations (**left**) and ResNet multi-channel input representation (**right**), derived from BLE (blue) and UWB (orange) anchors.

The key architectural distinction (Figure 4) lies in preserving per-anchor likelihoods as separate input channels rather than integrating through multiplication (Equation (8)). While the BGF multiplies likelihoods to obtain an integrated probability distribution for maximum likelihood estimation, the ResNet processes individual likelihood maps as distinct input channels. This enables the convolutional architecture to learn feature combinations and spatial patterns indicative of true position, including implicit weighting of anchor contributions based on propagation conditions and geometric configuration. These multi-channel likelihood representations serve as spatial feature inputs to the ResNet.

#### 2.4.2. ResNet Architecture

The ResNet architecture’s fundamental innovation lies in its skip connections, which enable identity mappings that support gradient flow through deep networks [60,61]. Each residual block implements the mapping $y=F(x,\left\{W_{\ell }\right\})+x$, where x represents the input to the block, F(·) denotes the learned residual mapping implemented through stacked convolutional layers, and $\left\{W_{\ell }\right\}$ represents the weights at each layer *ℓ* within the block.

**Figure 5:**
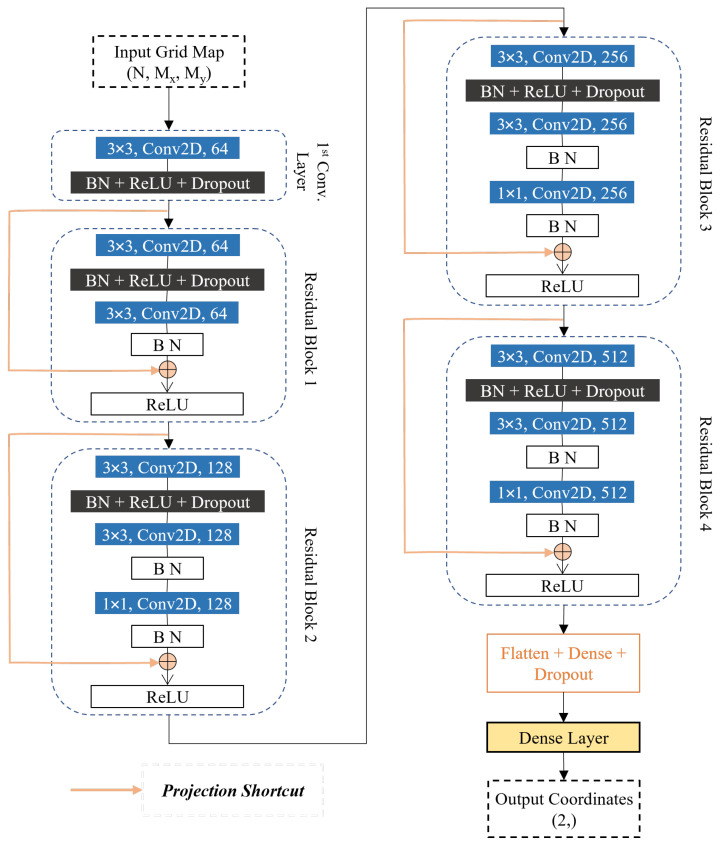
ResNet architecture diagram showing residual blocks, input dimensions (*N* channels for *N* anchors, each with $M_{x}\times M_{y}$ spatial resolution), and output layer producing 2D coordinates [22].

The hierarchical architecture employed for position estimation consists of four residual blocks with progressively increasing filter dimensions (64, 128, 256, 512 filters), enabling feature extraction at multiple scales from coarse spatial patterns in early layers to fine-grained positional information in deeper layers. Each residual block incorporates convolutional layers with batch normalization [62], rectified linear unit (ReLU) activation [63], and dropout regularization [64]. A flattening layer then aggregates the spatial features into a compact representation, and a fully connected dense layer with linear activation produces the final position estimate $\hat{p}={\left(\hat{x},\hat{y}\right)}^{⊤}$. The architectural implementation is illustrated in Figure 5.

The network input comprises stacked likelihood maps with dimensions $N\times M_{x}\times M_{y}$, where *N* denotes the number of anchors, and $M_{x}\times M_{y}$ represents the grid resolution. For the dual-technology system deployed in the cabin mockup, $N_{\mathrm{UWB}}=8$ UWB anchors and $N_{\mathrm{BLE}}=5$ BLE anchors provide complementary range measurements, yielding N=13 total input channels for data fusion. For scenarios with measurement failures from specific anchors, zero-valued arrays are substituted for the corresponding likelihood maps to maintain consistent input dimensionality. This approach enables the network to implicitly learn tolerance for missing measurements without requiring specialized handling mechanisms.

#### 2.4.3. Training Methodology and Validation Approach

We trained the ResNet model exclusively on real-world measurements from the experimental campaign described in Section 2.2. The training data comprised range measurements from the 19 surveyed tag positions within the cabin (Figure 3), with multiple measurement samples per position reflecting the stochastic nature of radio propagation. We partitioned the dataset into training (70%) and validation (30%) subsets, ensuring representative coverage of the spatial extent and varying measurement conditions. The model was trained to minimize the mean squared error (MSE) between predicted positions $\hat{p}$ and ground-truth positions p:

(10) $$L_{\mathrm{MSE}}=\frac{1}{B}\sum_{j=1}^{B}{∥{\hat{p}}_{j}-p_{j}∥}_{2}^{2}$$

where *B* denotes the batch size.

This loss function directly penalizes positioning errors in Euclidean space, aligning the optimization objective with practical positioning accuracy requirements [65]. Training employed the Adam optimizer [66] with hyperparameters selected through Bayesian optimization as detailed in Ammad et al. [22]. The architecture presented here extends the simulation-based proof of concept from prior work through training and validation with real-world measurements, capturing the full complexity of radio propagation, including multipath effects, NLOS conditions, and interference.

## 3. Results

### 3.1. Ranging Evaluation

The accuracy of radio-based localization systems fundamentally depends on the quality of the underlying observations. Prior to evaluating complete positioning performance, we analyze the ranging capabilities of both deployed technologies. Ranging residuals are computed for each measurement as the difference between the technology-reported distance and the true geometric distance derived from surveyed anchor and tag coordinates. Both radio systems provide raw ranging outputs, enabling assessment of the fundamental measurement characteristics that subsequent positioning algorithms must accommodate.

The analysis encompasses measurements from all 19 surveyed tag positions distributed throughout the cabin mockup, each measured at multiple height configurations (below, above and at seat level). For each tag position, ranging measurements to all visible anchor nodes were recorded, resulting in multiple range observations per position that reflect the varying propagation conditions across different anchor-tag constellations.

**Figure 6:**
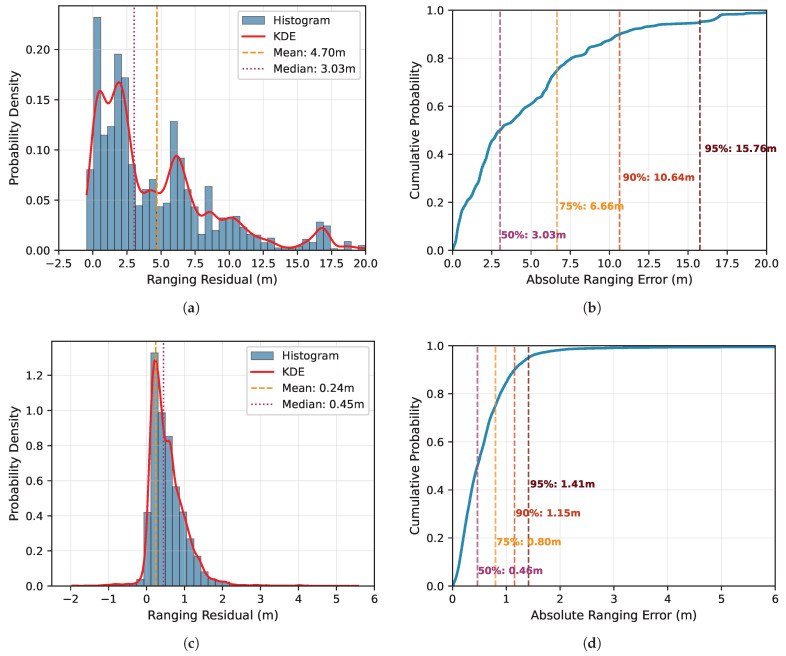
BLE and UWB ranging residual statistics displayed as histograms (left, bin width = 0.5 m, y-axis: relative frequency) and ECDFs (right, y-axis: cumulative probability): (**a**,**b**) BLE (99.1% of dataset shown) and (**c**,**d**) UWB (99.5% of dataset shown). The x-axis represents ranging residuals (measured minus true distance) in meters; axis limits differ to reflect the different residual ranges. Statistics are computed from the full datasets.

The complete dataset comprises several thousand ranging measurements for each technology, providing statistical significance for characterizing error distributions and identifying systematic biases. This characterization establishes baseline expectations for positioning accuracy given each technology’s measurement capabilities and identifies the error mechanisms and statistical properties that positioning algorithms must handle in the cabin environment. Figure 6 presents the statistical distribution of ranging residuals for both BLE and UWB measurements. The histogram representations (left panels) reveal the probability density of errors, while the empirical cumulated distribution function (ECDF) plots (right panels) enable quantitative assessment of percentile-based accuracy metrics.

The BLE system exhibits median ranging errors of 3.03 m, with 75% of measurements below 6.66 m. The UWB system demonstrates higher baseline accuracy, with median errors of 0.47 m and 95% of measurements within 1.41 m. Both distributions display pronounced right-skewed characteristics with extended positive tails, reflecting the error structure inherent to mixed LOS and NLOS propagation [20].

### 3.2. Positioning Performance

This section evaluates the positioning accuracy of four localization methods: ILS (Section 2.3.1), RLS (Section 2.3.2), BGF (Section 2.3.3), and ResNet-based positioning across individual technologies (BLE, UWB) and their fusion. All positioning errors are computed as two-dimensional Euclidean distances in the horizontal (x,y) plane. Height constitutes a controlled experimental variable affecting propagation conditions but is not estimated by the positioning algorithms. The analysis encompasses position estimates from 19 reference points distributed throughout the cabin mockup. The three baseline methods (ILS, RLS, BGF) are evaluated on all available measurement epochs. ResNet metrics are computed exclusively on the held-out validation set (30% of data, as described in Section 2.4) to report generalization performance on unseen samples.

**Figure 7:**
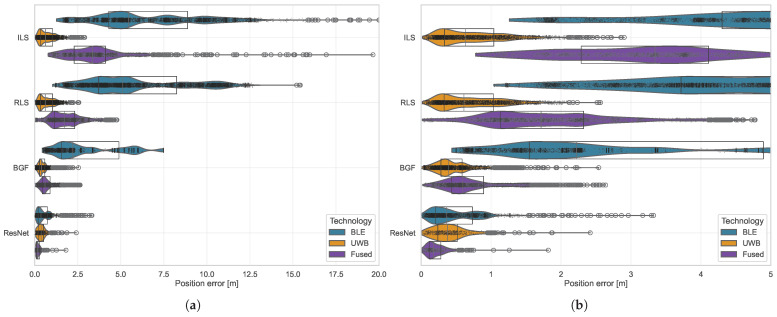
Positioning error distributions across methods and technologies. Raincloud plots combine violin plots, box plots (whiskers: 1.5× IQR), and individual data points. Panel (**a**) shows the complete error range; panel (**b**) provides detail for errors below 5 m. Methods are color-coded consistently across panels.

Figure 7 presents the positioning error distributions for all method–technology combinations through raincloud plots. The visualizations reveal systematic performance hierarchies across technologies, with UWB-based positioning consistently achieving lower median errors than BLE across all methods. ResNet demonstrated the lowest median errors within each technology category: 0.365 m (BLE), 0.376 m (UWB), and 0.177 m (Fused). Among traditional methods, grid-based filtering yielded median errors of 2.219 m (BLE), 0.391 m (UWB), and 0.595 m (Fused).

Table 1 summarizes positioning accuracy through statistical measures. The fusion approach achieved median performance intermediate between individual technologies (0.177 m for ResNet) while demonstrating reduced outlier frequency, as evidenced by the narrower spread between 75th and 95th percentiles (0.193 m for Fused ResNet versus 0.237 m for BLE- and 0.436 m for UWB ResNet). All method–technology combinations exhibited right-skewed error distributions, with mean errors exceeding median values by factors ranging from 1.03 (Fused ILS) to 1.38 (BLE BGF).

ILS showed pronounced sensitivity to outliers, with 95th percentile errors of 11.678 m (BLE), 1.593 m (UWB), and 6.184 m (Fused). RLS reduced these errors to 10.979 m, 1.523 m, and 3.745 m, respectively, representing improvements of 6% (BLE), 4% (UWB), and 39% (Fused). BGF further improved tail performance, with 95th percentile errors of 7.176 m (BLE), 1.143 m (UWB), and 1.645 m (Fused). ResNet achieved the lowest 95th percentile errors across all technologies: 0.969 m (BLE), 0.954 m (UWB), and 0.475 m (Fused).

**Figure 8:**
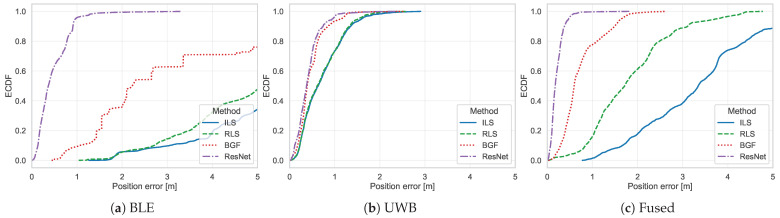
ECDFs of positioning errors (x-axis: error in meters; y-axis: cumulative probability) stratified by technology: (**a**) BLE, (**b**) UWB, (**c**) Fused. Each panel compares the four localization methods (ILS, RLS, BGF, ResNet), with line styles and colors indicated in the legend.

Figure 8 presents the ECDFs for all method–technology combinations. This analysis confirms that 75% of ResNet position estimates achieved errors below 0.732 m (BLE), 0.518 m (UWB), and 0.282 m (Fused). For BGF, the corresponding 75th percentile thresholds were 4.893 m (BLE), 0.586 m (UWB), and 0.893 m (Fused). ResNet achieved median positioning errors lower than BGF by factors of 6.1 (BLE), 1.0 (UWB), and 3.4 (Fused).

**Figure 9:**
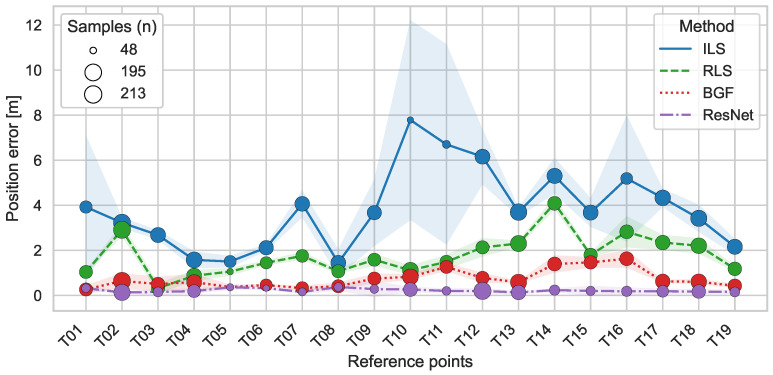
Mean positioning errors and standard deviations (error bars) per reference point for fused BLE and UWB. Reference point labels (T01–T19) correspond to the measurement locations described in Section 2.2. Marker size scales with sample count at each position.

Positioning accuracy exhibited substantial spatial variation across the reference points. Figure 9 displays the mean positioning error and standard deviation for each measurement location, stratified by method and technology. Central cabin positions along the aisle (T03, T04, T05, T08) consistently achieved mean errors below 1.5 m for traditional methods and below 0.3 m for ResNet across all technology configurations. Peripheral positions near window seats (T10, T11, T15, T16) demonstrated elevated errors exceeding 3.0 m for parametric methods, with standard deviations indicating high within-position variability.

BGF maintained more spatially uniform performance than parametric methods, with inter-position standard deviation of 0.54 m (based on position-wise means) compared to 1.24 m for RLS and 1.87 m for ILS in the fused configuration. Window-seat positions consistently exhibited higher errors than aisle positions across all parametric methods, while ResNet demonstrated reduced spatial performance variation. Measurement height influenced positioning accuracy through anchor visibility and LOS conditions. Elevated positions approximately 1 m above the cabin floor (T11, T12, T16) provided improved visibility to wall-mounted anchors, with BGF achieving 18% lower mean errors compared to seat-level measurements. Parametric methods demonstrated minimal height sensitivity, suggesting reduced reliance on LOS/NLOS classification. Floor-level measurements (T19) yielded intermediate positioning errors, with the highest variability for parametric approaches.

## 4. Discussion

### 4.1. Ranging Characteristics and Error Mechanisms

The right-skewed ranging residual distributions (Section 3.1) reflect mixed LOS/NLOS propagation inherent to the aircraft cabin environment. Both technologies exhibit asymmetric error patterns. A dominant mode near zero corresponds to LOS measurements affected primarily by thermal noise and hardware limitations, while extended positive tails capture NLOS-induced biases from signal reflection and diffraction through cabin structures. BLE’s median ranging error of 3.03 m versus UWB’s 0.47 m correlates with bandwidth limitations [20,45]. These positive tails arise from technology-specific NLOS mechanisms. For UWB, first-path detection failures under signal attenuation yield systematic positive biases reflecting reflected/diffracted propagation paths [20]. BLE’s narrower bandwidth limits temporal discrimination of closely-spaced multipath components, creating constructive/destructive interference across frequency channels that degrades phase measurement accuracy. Beyond random measurement errors, spatial correlation between NLOS conditions and cabin geometry creates position-dependent error patterns: specific anchor-tag configurations consistently exhibit degraded ranging due to obstruction.

These asymmetric, non-Gaussian error distributions violate optimality assumptions of least-squares and Kalman filtering approaches, introducing systematic position biases when NLOS measurements dominate [53]. Robust M-estimators provide partial mitigation [54], but cannot fully compensate for systematic biases or spatial error correlation, which explains the persistent performance gap between RLS and BGF observed in Section 3.2.

### 4.2. Generalization and Training Data Characteristics

The ResNet evaluation employed an independent validation set (30% of data, n=652 for fused configuration) unseen during training, ensuring reported performance metrics in Table 1 reflect genuine generalization rather than memorization. The architecture incorporates dropout regularization and batch normalization to mitigate overfitting. Consistent performance across spatially diverse positions (aisle, window, different heights) indicates that the network learns generalizable spatial patterns rather than memorizing specific measurement instances. The lower ResNet positioning error (median 0.177 m fused vs. 0.595 m for BGF) demonstrates effective learning from the presented measurement campaign.

The trained model is specific to the A320 cabin configuration in which data was collected: the network learns spatial patterns from likelihood maps that encode the particular anchor geometry, propagation characteristics, and environmental reflections of this cabin. Deploying the model in a different aircraft configuration would require retraining with environment-specific data. However, the methodology of transforming range measurements into spatial likelihood maps and processing them with a convolutional architecture is geometry-agnostic and transferable to arbitrary environments with different anchor deployments and cabin geometries.

The stationary measurement conditions further constrain the diversity of error patterns encountered during training. This environmental consistency likely contributes to the observed accuracy gains, as the bounded error space enables reliable pattern recognition with limited spatial samples. Operational deployment introduces variability absent from the training distribution: body-worn devices exhibit orientation-dependent antenna patterns, passenger movement creates time-varying shadowing, and cabin occupancy alters propagation through human tissue absorption and scattering [14]. The network’s learned spatial representations may not generalize reliably to these out-of-distribution conditions. Future validation should assess performance degradation under dynamic tracking scenarios, varying passenger densities (empty vs operational load factors of 80–90%), and different aircraft configurations to establish practical deployment bounds.

### 4.3. Spatial Performance Heterogeneity and Learned Compensation

Spatial heterogeneity in Figure 9 reflects coupled geometric and propagation mechanisms. The elongated cabin geometry with longitudinal anchor placement creates anisotropic geometric dilution of precision (GDOP), with window-seat positions (T06, T07, T10, T17, T18) experiencing reduced anchor diversity and increased NLOS probability from seat structures. This resulted in 62% higher errors for ILS compared to aisle positions. ResNet’s reduction in this window-aisle performance gap to 28% demonstrates learned compensation for position-dependent error patterns that geometric methods cannot accommodate.

The convolutional architecture learns spatial patterns from likelihood maps that encode geometric positioning constraints, which remain valid across different anchor configurations. The pronounced height sensitivity (elevated positions achieving 18% lower errors for BGF, Figure 9) directly correlates with improved LOS to wall-mounted anchors at approximately 1.9 m height. BGF’s explicit probabilistic likelihood evaluation (Equation (8)) directly rewards reduced ranging residuals from LOS measurements, while ResNet’s reduced height sensitivity suggests implicit learned NLOS compensation.

BGF maintained higher spatial consistency by preserving discrete probability distributions rather than collapsing to parametric point estimates, enabling reliable handling of multimodal likelihoods under severe multipath conditions [57].

### 4.4. Algorithmic Resilience to Non-Gaussian Errors

The non-Gaussian error characteristics described in Section 4 affect the four positioning methods differently. The persistent performance gap between RLS (median 1.714 m) and BGF (0.595 m) for the fused configuration demonstrates that linear down-weighting of outliers cannot fully compensate for the systematic positive biases introduced by NLOS propagation. BGF’s non-parametric likelihood evaluation preserves the full error structure without distributional assumptions, enabling more accurate position estimation under mixed LOS/NLOS conditions. ResNet’s learned representation implicitly captures these complex error characteristics through spatial correlation patterns in likelihood maps, achieving the lowest positioning error (median 0.177 m) without explicit NLOS modeling or predefined loss functions.

### 4.5. Baseline Selection and Architectural Considerations

Various deep learning architectures have been proposed for indoor positioning, including LSTMs for trajectory tracking, MLPs for raw signal processing, and attention-based mechanisms for channel state information [9,10,12]. However, the proposed methodology transforms raw range measurements into spatial likelihood maps, framing the positioning problem as a computer vision task.

This image-based spatial representation makes convolutional neural networks the natural architectural family. ResNet was selected as a representative CNN backbone not to claim architectural superiority over all deep learning models but to validate likelihood-based CNN processing in the multipath-rich aircraft cabin environment.

The baseline methods (ILS, RLS, BGF) represent the current state-of-the-practice in range-based positioning for industrial and aviation deployment. The primary objective of this study is to quantify the performance gap between these traditional geometric and probabilistic methods and data-driven deep learning approaches. In the context of aviation, where interpretability, certification requirements, and established baselines guide technology adoption, these geometric solvers represent a critical benchmark. The methodological progression from ILS (parametric) through RLS (robust parametric) to BGF (non-parametric probabilistic) and finally ResNet (data-driven) isolates the methodological contribution and demonstrates whether deep learning offers a statistically significant advantage in the metallic cabin environment. The open publication of the complete measurement dataset [67,68] enables the research community to evaluate alternative deep learning architectures—including Transformers, attention mechanisms, and graph neural networks—against the empirical baselines established here. We view this dataset contribution as an explicit invitation for architectural comparison studies that extend beyond the scope of this application-focused validation. Regarding computational feasibility, the ResNet architecture comprises approximately 11.2 million trainable parameters across four residual blocks (64, 128, 256, 512 filters). Likelihood computation for N=13 anchors on the $M_{x}\times M_{y}$ grid constitutes the primary computational cost, requiring $O(N\cdot M_{x}\cdot M_{y})$ distance evaluations per update. On standard hardware, complete processing from raw measurements to position estimate requires approximately 50 ms per sample, well within the requirements of cabin positioning applications where the UWB measurement system operates at approximately 10 Hz, providing a measurement update budget of 100 ms.

### 4.6. Limitations and Future Validation Requirements

Several experimental constraints limit generalization to operational scenarios. The measurements used reference-grade UWB tags and dedicated BLE hardware for controlled evaluation; in operational deployment, consumer devices (smartphones with UWB and BLE, or card form factor tags) would serve as mobile nodes.

The tag form factor does not affect the measurement physics or algorithm design, as the positioning methods process range observations identically regardless of hardware origin, though consumer-grade devices may exhibit different noise characteristics. The empty cabin represents best-case conditions absent the tissue absorption, body shadowing, and time-varying scattering from passengers [14,33]. Static tag positioning eliminated motion-induced variability but did not capture device orientation effects or body-worn deployment conditions encountered in operational passenger tracking. The 19 surveyed positions provide limited spatial sampling, with measurements concentrated in a 10-seat-row section leaving forward/aft regions uncharacterized. Single-point-in-time measurements preclude assessment of system stability or anchor drift over extended operational periods. Validation restricted to narrow-body A320 configuration precludes generalization assessment across wide-body or regional varying cabin geometries and structural compositions. These constraints are shared across all empirical aircraft cabin positioning studies. Karadeniz et al. [36] reported results from a single environment, Neuhold et al. [35] used a single-section test setup, and Geyer and Schupke [37] tested in one cabin configuration, none with open data publication. With 19 geodetically surveyed positions, dual-technology ranging data, and open-access dataset publication, the present study forms one of the most extensive empirical efforts in aircraft cabin positioning to date.

Future work should prioritize validation under realistic operational conditions to address the identified limitations. Planned measurement campaigns will evaluate dynamic passenger tracking scenarios with crowded cabins approximating operational load factors, extended spatial coverage spanning complete cabin length, and multi-aircraft validation across narrow-body and wide-body configurations.

Transfer learning offers a promising strategy for extending the validated approach to other aircraft types without requiring full measurement campaigns. The likelihood maps provide a natural foundation for domain adaptation, as their spatial structure remains consistent across environments, while only the anchor geometry and propagation characteristics change. Models pre-trained on one cabin configuration can be fine-tuned with limited data from a target environment, reducing the required measurement effort. Wang et al. [69] demonstrated deep domain adaptation for indoor localization across different environments, and Zholamanov et al. [70] showed that transfer learning effectively addresses the domain shift between different indoor environments for fingerprint-based localization. Simulation-based pre-training via deterministic ray-tracing of target cabin geometries [19] could provide synthetic likelihood maps for initial model training, with subsequent fine-tuning using limited real-world measurements in a sim-to-real transfer approach. The publication of the entire dataset as an open-access resource [67,68] enables the research community to benchmark alternative deep learning architectures—including Transformers, attention-based mechanisms, and other emerging approaches—against the baseline results presented in this study. By providing raw range measurements alongside surveyed ground-truth positions, the dataset supports direct comparison of both positioning algorithms and input representations, establishing a reusable benchmark for aircraft cabin localization research.

## Figures and Tables

**Table 1:** Positioning performance comparison across technologies and methods (metrics in meters).

Technology	Method	Samples	Mean	Median	Std Dev	P75	P95
BLE	ILS	7500	6.569	5.459	2.990	8.881	11.678
	RLS	11,166	5.806	5.114	2.832	8.243	10.979
	BGF	11,166	3.057	2.219	1.953	4.893	7.176
	ResNet	3353	**0.471**	**0.365**	**0.355**	**0.732**	**0.969**
UWB	ILS	4395	0.726	0.635	0.473	1.039	1.593
	RLS	4399	0.706	0.612	0.444	1.032	1.523
	BGF	4399	0.469	0.391	0.296	0.586	1.143
	ResNet	660	**0.416**	**0.376**	**0.272**	**0.518**	**0.954**
Fused	ILS	3414	3.437	3.344	1.671	4.108	6.184
	RLS	4101	1.832	1.714	0.918	2.323	3.745
	BGF	4101	0.739	0.595	0.447	0.893	1.645
	ResNet	652	**0.212**	**0.177**	**0.152**	**0.282**	**0.475**

P75 and P95 denote the 75th and 95th percentiles, respectively. Lowest error per technology is indicated in bold for reference. Sample counts differ across methods: ILS processes fewer epochs than RLS and BGF because the Gauss–Newton iteration may fail to converge for measurement configurations with poor geometry. ResNet sample counts reflect evaluation on the held-out validation set (30%) only, whereas baseline methods are evaluated on all available epochs.

## Data Availability

The data presented in the study is openly available at Zenodo [67,68].

## Abbreviations

The following abbreviations are used in this manuscript:

AI	Artificial intelligence
API	Application programming interface
BGF	Bayesian grid filter
BLE	Bluetooth Low Energy
CNN	Convolutional neural networks
ECDF	Empirical cumulative distribution function
GDOP	Geometric dilution of precision
ILS	Iterative least squares
IPS	Indoor positioning systems
ISM	Industrial, scientific and medical band
LOS	Line-of-sight
LSTM	Long short-term memory
ML	Machine Learning
MSE	Mean Squared Error
NLOS	Non-line-of-sight
ReLU	Rectified Linear Unit
ResNet	Residual neural network
RLS	Robust least squares
TWR	Two-way ranging
UWB	Ultra-Wide Band
WiFi	Wireless fidelity
